# Early adherence to antihypertensive drugs and long‐term cardiovascular mortality in the “real world”

**DOI:** 10.1111/jch.14319

**Published:** 2021-07-13

**Authors:** Martin R. Salazar

**Affiliations:** ^1^ Facultad de Ciencias Médicas Universidad Nacional de La Plata (UNLP) Calle 1 y 70 La Plata Argentina 1900 Argentina; ^2^ Cátedra de Medicina Interna Hospital San Martín La Plata Argentina; ^3^ Sociedad Argentina de Hipertensión Arterial (SAHA) Buenos Aires Argentina

High blood pressure is the leading cause of death and disability worldwide and in this number of the Journal of Clinical Hypertension, Cho‐Long Kim and colleagues apport new insights to this relevant issue.^1^ It has been estimated that more than 200 million of disability adjusted life years (DALYs) were lost every year because of blood pressure above the optimal levels.^2^ Fortunately, pharmacological treatments effectively reduce the risk of cardiovascular events and decrease morbidity and mortality. However, non‐adherence is a significant barrier to effective blood pressure reduction in the “real world”.^3^, ^4^ Nonadherence to antihypertensive treatment affects 10–80% of hypertensive patients and is one of the key drivers of suboptimal blood pressure control.^5^ A population‐based cohort study including more than 100 000 patients (using the United Kingdom General Practice Research Database) found that overall antihypertensive drug discontinuation was ∼20% at 6 months and ∼30% at 1 year.^6^ Furthermore, retrospective cohort information from 320 Italian general practitioners including 13 000 patients showed that ∼40% discontinued their treatment at 1 year.^7^ Thus, adherence to antihypertensive medication is an important challenge that doctors often face in the treatment of hypertension.

Poor adherence to antihypertensive treatment correlates with the magnitude of BP elevation and is an indicator of poor prognosis in hypertensive patients.^8^ In a cohort of 250 000 newly treated patients with hypertension from the Italian Lombardy Region, those who continued treatment had a 37% reduced risk of cardiovascular disease compared with patients who experienced at least one episode of treatment discontinuation.^9^ However, long‐term populational data analyzing the relationships between non‐adherence and cardiovascular mortality are scanty. To evaluate the long‐term effects of early antihypertensive medication adherence, Cho‐Long Kim and colleagues^1^ analyzed the relationships between medication adherence and cardiovascular mortality in a retrospective cohort of 20 836 patients with newly diagnosed hypertension. Data were extracted from the Korean National Health Insurance Service (NHIS). Adherence was estimated using the compliance ratio (CR) during the first year after the diagnosis of hypertension. Additionally, the cohort was stratified according to the presence or absence of cardiovascular complications. Individuals with cardiovascular disease were ∼5 years older and had a higher prevalence of hypertension and diabetes mellitus. During an observation period, 2,7% died due to cardiovascular disease. One year after diagnosis of hypertension, 54.9% had a CR ≥70%. In the patients without complications, the risk of cardiovascular death was significantly lower with CR ≥70% (hazard ratio, 0.56; *p* = .014). However, in patients with cardiovascular disease, there was no significant difference in risk of cardiovascular death between CR ≥70% and CR < 70% groups. Only with CR ≥90%, a lower risk of cardiovascular mortality was observed (hazard ratio, 0.56; *p* < .001). Thus, the authors concluded: “Medication adherence is significantly associated with cardiovascular mortality during 10 years in newly diagnosed hypertensives patients. Patients with complications of hypertension have to continue a high adherence rate (CR≥90) for better long‐term clinical outcomes. The results of this study are consistent with those of previously published studies. Mazzaglia G and colleagues, using data of 18 806 newly diagnosed hypertensive patients from 400 Italian primary care physicians, showed that high adherers had (proportion of days covered, ≥ 80%) a significantly decreased risk of acute cardiovascular events (hazard ratio, 0.62; 95% CI, 0.40–0.96).^10^ The study by s Cho‐Long Kim and colleagues^1^ expands the conclusion to cardiovascular mortality.

The barriers to optimal adherence may be linked with physician attitudes, patient beliefs, and behavior, the complexity and tolerability of drug therapies, the health care system, and several other factors. Burnier et al., identify two different ways: (1) short persistence when the patients ceased their engagement with the dosing regimen on their initiative, an inherently willful act, not arising from forgetfulness., and (2) lapses in implementation (or execution) because of forgetfulness or negligence.^3^ The barriers include some causes associated with the health care system and socioeconomic status factors. In consequence, the prevalence of non‐adherence varies widely worldwide.^11^ A meta‐analysis reported that studies carried out in Africa showed lower adherence levels than Asians, Europeans, and Americans studies.^12^ In Argentina, we found than 50% of treated hypertensive patients had a high level of adherence. These patients had lower BP values and higher control levels. Interestingly, high educational level was an independent and strong predictor for high adherence.^13^

Patient adherence to therapy can be improved by several interventions and multicomponent interventions could have a greater effect on adherence, as the effect size of each intervention is generally modest. Health care system interventions such as the development of monitoring systems and national databases, including prescription data are necessary. Thus, “real world” studies designed to identify regional or national characteristics of non‐adherence should be encouraged. Data obtained from these studies could be critically important to design local preventive campaigns.

The study by Cho‐Long Kim and colleagues^1^ has some interesting findings that should be highlighted. Firstly, early high adherence levels decrease long‐term cardiovascular mortality, supporting the importance of early blood pressure control. Early discontinuation of treatment is a common facet of poor adherence. Moreover, early recognition of a lack of adherence might reduce further investigations and avoid the prescription of unnecessary drugs. A meta‐analysis of data on 376 162 patients from 20 studies assessing adherence using prescription refill frequency showed that after 6 months more than 30%, and after 1 year, about 50% of patients may stop their initial treatment.^14^ This study suggests that more than 70% of prescribed doses are necessary to decrease cardiovascular mortality. Secondly, patients who developed complications of hypertension during the follow‐up period need a more intensive level of adherence to antihypertensive medication than those without complications. This finding is consistent with the proposed stricter blood pressure goals for secondary prevention of cardiovascular disease. Finally, the study evaluated a hard end point, cardiovascular mortality.

On the other hand, the study has some drawbacks. Firstly, it is a retrospective analysis from the database of the Korean NHIS and no causality could be established. Secondly, the study did not use medical recordings. In consequence, patient factors such as psychological factors and level of knowledge of disease were not considered. Moreover, there could be some bias in the diagnosis of cardiovascular complications because of missing data. Thirdly, the results could not extrapolate to other populations or ethnics groups. Finally, the way how adherence was evaluated in this study is a debatable issue. To estimate the level of adherence, direct and indirect methods could be used. Today, the most accurate method is the detection of prescribed drugs in blood or urine samples. However, although the direct methods have greater accuracy, the high cost and lack of availability have limited their use. Thus, indirect methods are more frequently used in both, daily medical practice, and epidemiological studies. The authors used the CR to evaluate the adherence, a modified version of the proportion of days covered (PDC). CR is based on electronic pharmacy records and does not use patient data. Thus, since only medication dispensing records were used, the patient's actual adherence to antihypertensive drugs can be different. I think that this is the main limitation of the study. However, the PDC showed a relationship with cardiovascular mortality in a population‐based, observational, longitudinal study performed in a large cohort of AMI survivors from Canada.^15^

In conclusion, despite their inherent limitations the study conducted by Cho‐Long Kim and colleagues apports valuable evidence regarding the critical importance of early adherence to antihypertensive drugs to decrease long‐term cardiovascular mortality, particularly in patients with established cardiovascular disease.

## CONFLICT OF INTEREST

None.
